# The Medical Profession in Relation to Inquests

**Published:** 1911-12-02

**Authors:** 


### THE MEDICAL PROFESSION IN RELATION TO INQUESTS.

Some disturbance was caused in the minds of many medical men a few years ago by the action of a coroner in employing a pathologist to perform post-mortem examinations to the exclusion of the medical man who had seen the deceased person during life. There was much to be said upon both sides of the controversy which arose, and there is perhaps much to be said to-day. The control of much that has relation to disease has during comparatively recent years passed into the hands of paid officers of public authorities. Although this, no doubt, in some ways is to the advantage of the community, the practitioner suffers pecuniary loss. Naturally, therefore, he is not inclined to view any further deprivation of fees with favour, and unless the deprivation appeals to his sense of justice it is likely to meet with his animosity. Here it may be said that there are grounds for believing that to employ a pathologist to the exclusion of the medical man who saw the case during life does seem unreasonable. There are many cases seen in the post-mortem room in which in the absence of knowledge of symptoms occurring during life it would be impossible for a pathologist to state definitely the cause of death. The medical man who has seen the case during life should always be called in to give evidence, but the post-mortem examination should be made by someone of experience.

This would increase the expenses entailed in the work of a coroner, but need not necessarily do so to any great extent. In these days it is true that every medical student is supposed to gain some experience of work in the post-mortem room, but how small is this experience in most instances, and how quickly is the greater part of the knowledge of the appearance of disease lost as soon as opportunities for retaining it cease to exist. Many instances may be cited to support the opinion that a medical man in practice has very insufficient knowledge of morbid anatomy.

We do not intend to imply that the opinion of a man of experience is infallible. Most of us, it is to be feared, are very superficial ourselves. Dr. Moxon long ago remarked that "we only retain as knowledge what has been an answer to a question in our own minds." However great the experience, much may be overlooked unless in some way attention has been drawn to a point in question. If post-mortem evidence in cases likely to become medico-legal cases is to be of value, the one gaining his experience must have his powers of observation continually stimulated by inquiry. A man may have considerable experience, but appearances are so varied that his attention may not be called to some point of apparently small importance which may prove to be of great importance in some particular case. Medico-legally the experience of many thousands of cases is scattered through many hundreds of minds, and is of little use to anyone, because in every instance it is too scrappy.

Allowing that the establishing of some such system is desirable, a question arises as to the source from which the pathologists are to be drawn. Those medical men who hold the appointment of police surgeon may in some instances desire to engage in the work, but this is not likely frequently to be the case. Medical men in private practice will rarely be able to spend the greater part of their time in connection with inquests. On the other hand, to obtain a sufficient number of morbid anatomists not engaged in general practice to perform duties required by coroners may not be easy. To suggest that post-mortem examinations should be reduced in number would in some districts possibly affect medical men. What may happen in the future with regard to examination of the body after death from the point of view of investigation of disease is another question. Possibly it might be well if this were carried out as a matter of routine. However this may be, there can be no doubt that in many cases evidence given before the coroner upon knowledge of the case during life is sufficient.

